# Supplementary material for: The brain dynamics of architectural affordances during transition
Source: Sci Rep. 2021 Feb 2;11:2796. doi: 10.1038/s41598-021-82504-w (PMC7854617; doi:10.1038/s41598-021-82504-w)

Supplementary Figures for:

The neuroscience of liminality in architectural passages

Zakaria Djebbara[1*], Lars Brorson Fich[1], Klaus Gramann[2,3,4]

[1] Aalborg University, Department of Architecture, Design and Media Technology
[2] Biological Psychology and Neuroergonomics, Berlin Institute of Technology
[3] School of Software, University of Technology Sydney
[4] Center for Advanced Neurological Engineering, University of California
*Corresponding author: zadj@create.aau.dk

Data accessibility:
https://osf.io/xywdh/

Supplementary material 1; 1 of 4 (*immobile phase*)

'The neuroscience of liminality in architectural passages'.

Time-frequency analyses of all generated clusters and belonging topographical plot. As cluster 1 and 2 were the parental cluster and the outlier cluster, respectively, they have been omitted from the list below.
Furthermore, clusters that did reach significance (ANOVA) are emphasized with a red frame.

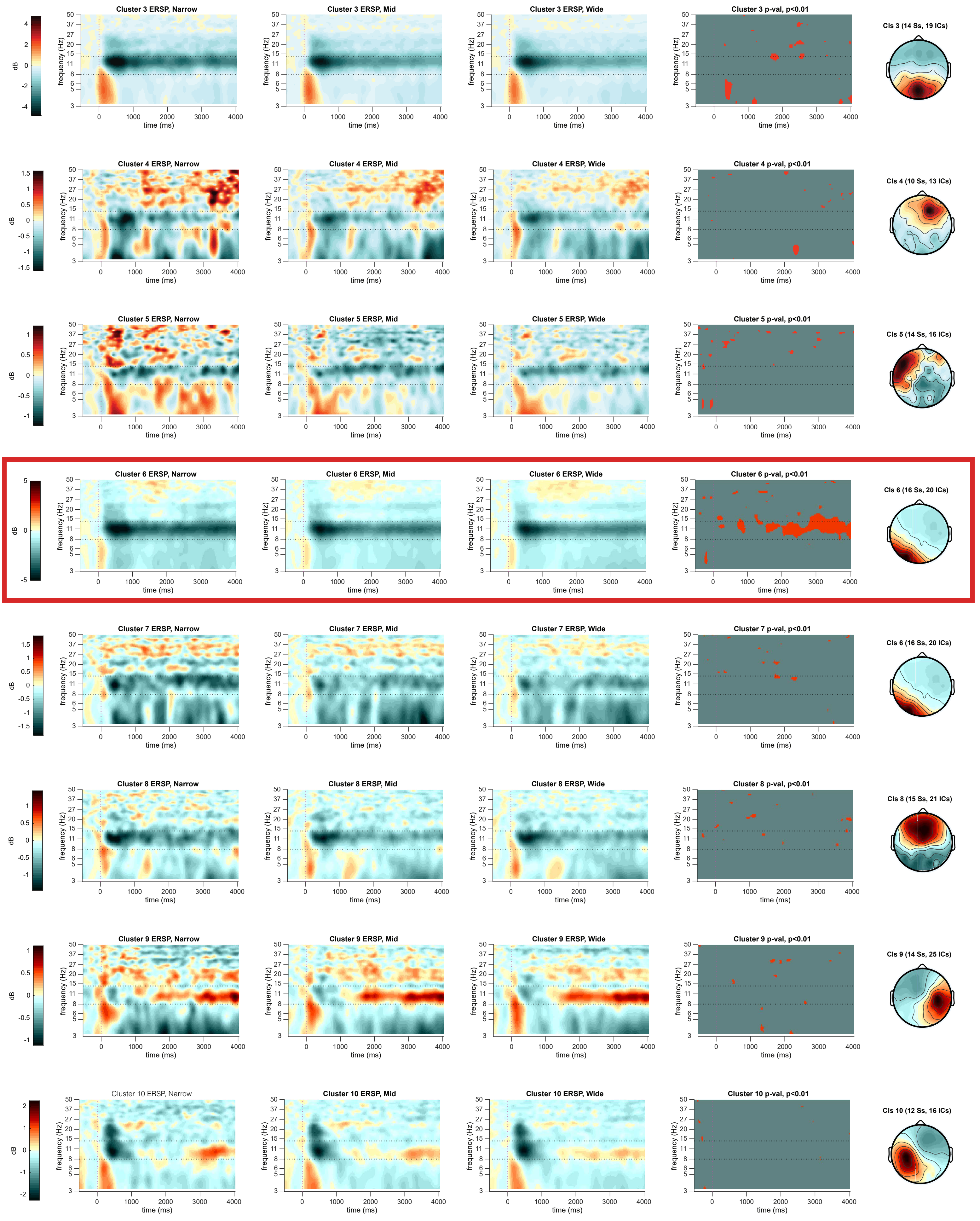

Supplementary material 1; 2 of 4 (*immobile phase*)

'The neuroscience of liminality in architectural passages'.

Time-frequency analyses of all generated clusters and belonging topographical plot. As cluster 1 and 2 were the parental cluster and the outlier cluster, respectively, they have been omitted from the list below.
Furthermore, clusters that did reach significance (ANOVA) are emphasized with a red frame.

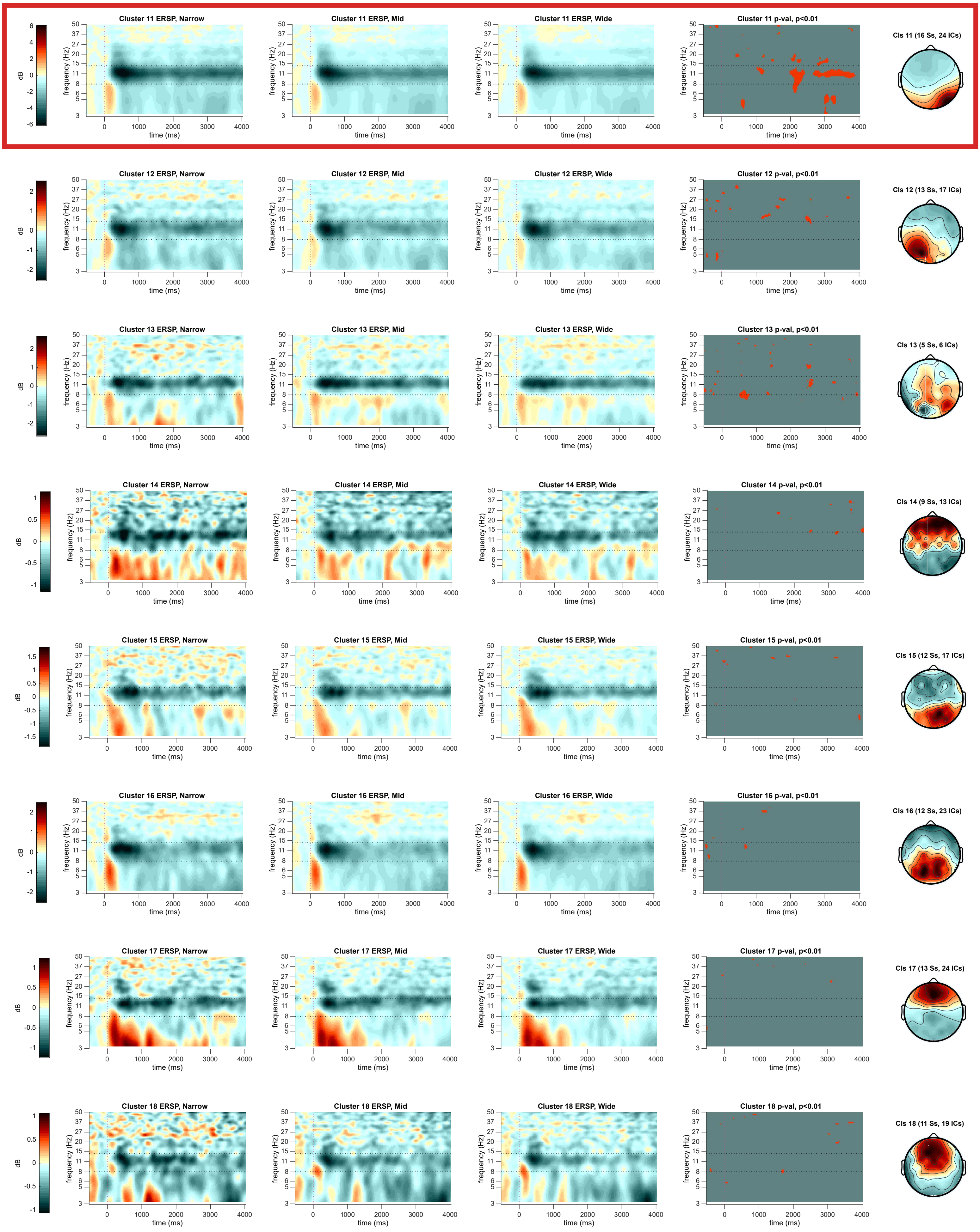

Supplementary material 2; 3 of 4 (*mobile phase*):

'The neuroscience of liminality in architectural passages'.

Time-frequency analyses of all generated clusters and belonging topographical plot. As cluster 1 and 2 were the parental cluster and the outlier cluster, respectively, they have been omitted from the list below. Furthermore, clusters that did reach significance (ANOVA) are emphasized with a red frame.

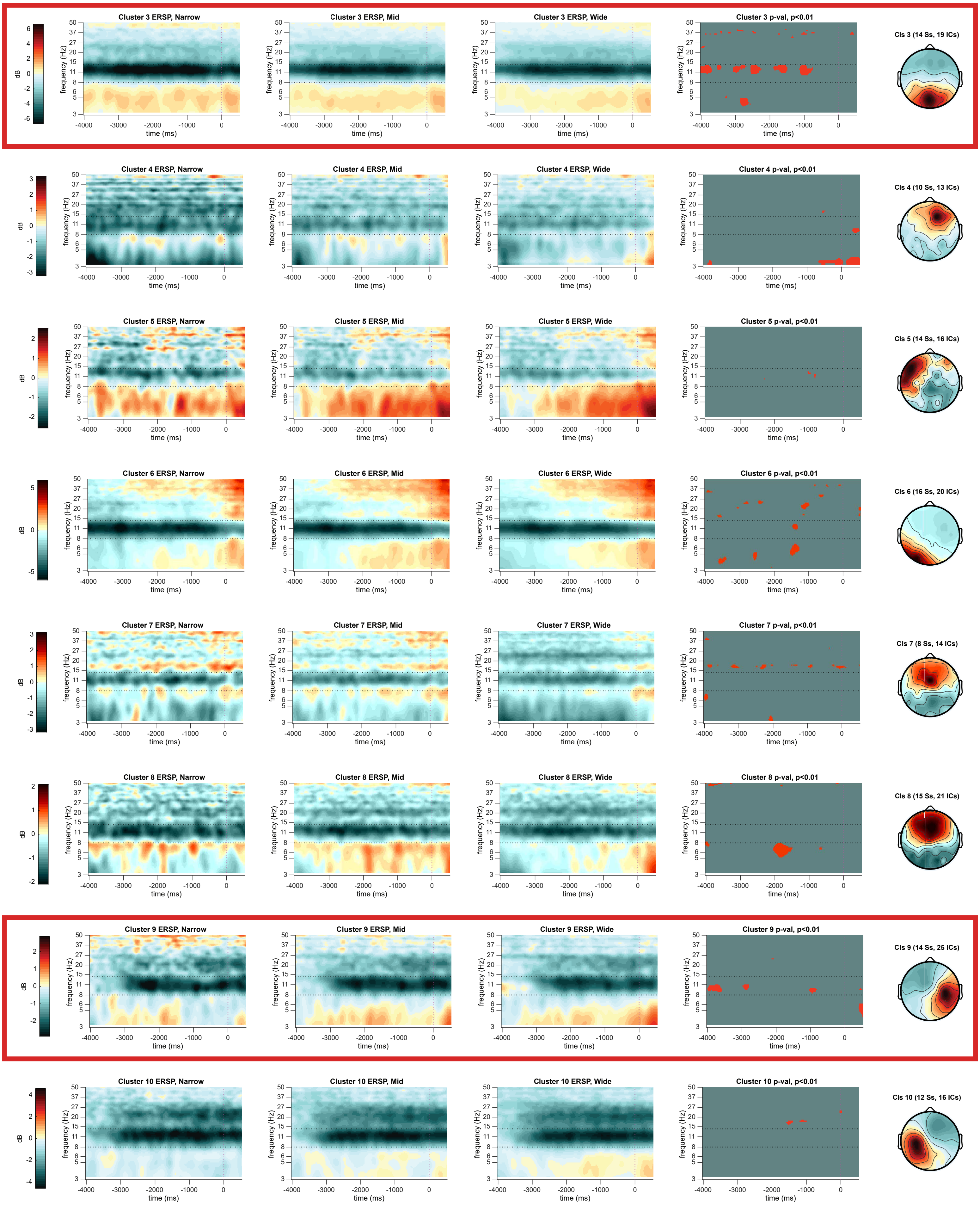

Supplementary material 2; 4 of 4 (*mobile phase*):

'The neuroscience of liminality in architectural passages.

Time-frequency analyses of all generated clusters and belonging topographical plot. As cluster 1 and 2 were the parental cluster and the outlier cluster, respectively, they have been omitted from the list below. Furthermore, clusters that did reach significance (ANOVA) are emphasized with a red frame.

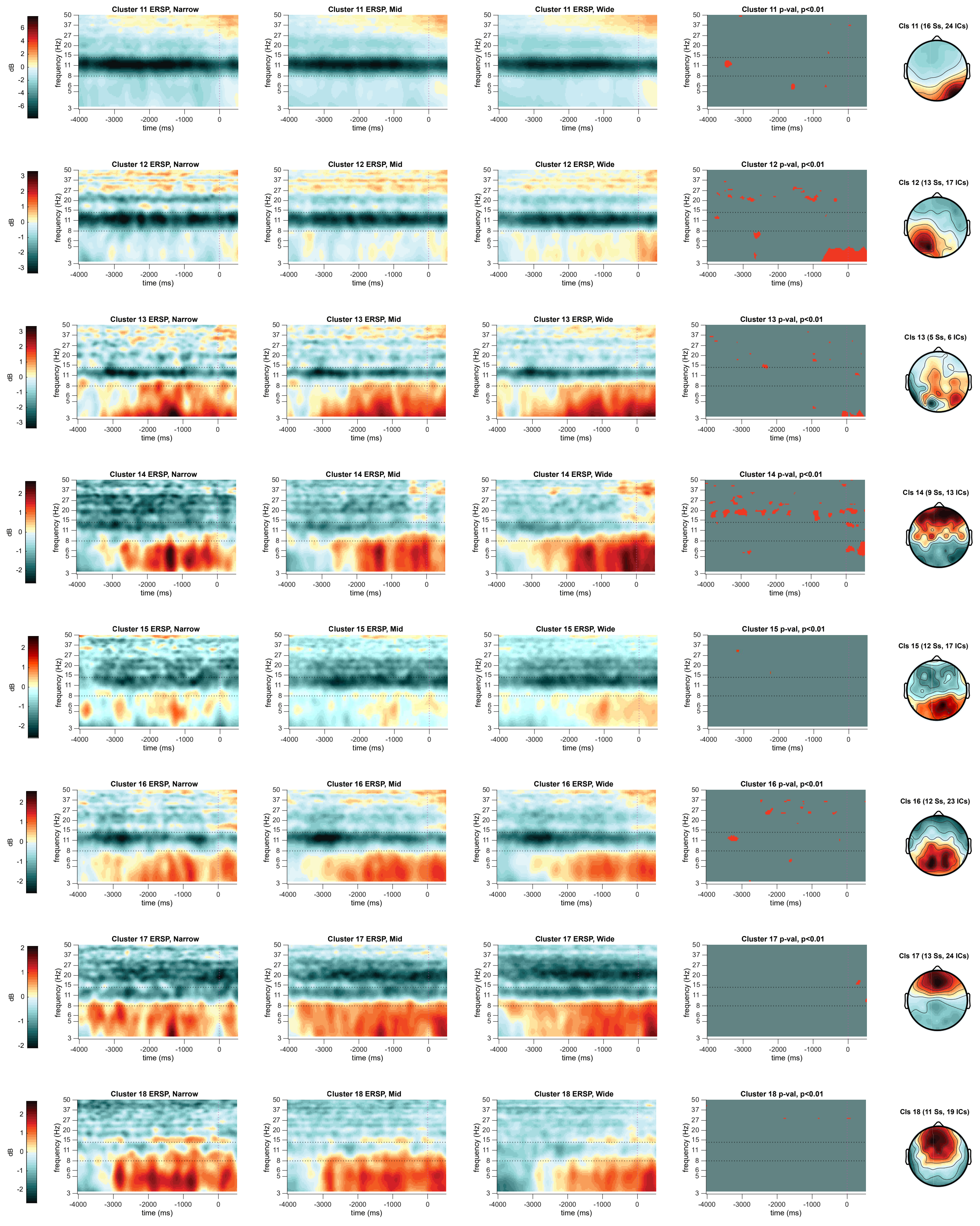

Supplement: Supplementary file 1 — Supplementary figures [file 41598_2021_82504_MOESM1_ESM.pdf]
